# Diagnostic accuracy of circulating microRNAs and lipidomic biomarkers for early breast cancer detection: A systematic review and meta-analysis

**DOI:** 10.1371/journal.pone.0354728

**Published:** 2026-07-24

**Authors:** Heba Mohammed Arafat, Akbar Ali, Tengku Ahmad Damitri Al Astani Tengku Din, Ohood Mohammed Shamallakh, Rashid Jusoh, Maya Mazuwin Yahya, Wan Zainira Wan Zain, Wan Faiziah Wan Abdul Rahman

**Affiliations:** 1 Department of Chemical Pathology, School of Medical Sciences, Health Campus, Universiti Sains Malaysia, Kubang Kerian, Kelantan, Malaysia; 2 Breast Cancer Awareness and Research Unit, Hospital Pakar Universiti Sains Malaysia, Health Campus, Kubang Kerian, Kelantan, Malaysia; 3 Department of Medical Laboratory Sciences, Faculty of Health Sciences, Islamic University of Gaza, Gaza City, Palestine; 4 Department of Surgery, School of Medical Sciences, Universiti Sains Malaysia, Health Campus, Kubang Kerian, Kelantan, Malaysia; 5 Department of Pathology, School of Medical Sciences, Universiti Sains Malaysia, Health Campus, Kubang Kerian, Kelantan, Malaysia; University of Diyala College of Medicine, IRAQ

## Abstract

**Background:**

Breast cancer outcomes improve substantially with earlier detection, yet mammography performance can be limited in dense breasts and may lead to false-positive investigations. Circulating microRNAs (miRNAs) and lipidomic/metabolomic signatures have emerged as promising minimally invasive biomarkers that could complement imaging for early-stage detection. The aim of this systematic review and meta-analysis is to evaluate the diagnostic accuracy of circulating microRNAs and lipidomic/metabolomic biomarkers for early breast cancer detection and to explore between-study heterogeneity.

**Materials and methods:**

A PRISMA 2020–compliant systematic review and meta-analysis were conducted. MEDLINE (PubMed), Web of Science, Scopus, Springer, ScienceDirect, and the Cochrane Library were searched for eligible diagnostic studies assessing circulating microRNAs and lipidomic/metabolomic biomarkers measured in serum or plasma. No language restrictions were applied. Non-English reports were screened and, when potentially eligible, translated for full-text assessment and data extraction. Studies using whole blood were excluded. Study quality was assessed using QUADAS-2. Random-effects models (DerSimonian–Laird) pooled sensitivity, specificity, likelihood ratios, diagnostic odds ratio (DOR), and summary ROC (SROC) area under the curve (AUC). Heterogeneity was evaluated using Q and I^2^ statistics, and small-study effects were assessed using Deeks’ test.

**Results:**

Thirty-two studies (2015–2025) comprising 6,935 participants (3,697 breast cancer cases; 3,238 controls) were included. Pooled sensitivity was 0.87 (95% CI [0.83, 0.90]) and pooled specificity was 0.84 (95% CI [0.79, 0.88]), with pooled DOR 46.10 (95% CI [27.80, 76.70]), PLR 5.17 (95% CI [3.99, 6.68]), NLR 0.16 (95% CI [0.13, 0.21]), and SROC AUC 0.92 (95% CI [0.87, 0.94]). Heterogeneity was substantial (I^2^ = 88.29% for sensitivity; I^2^ = 90.20% for specificity). In subgroup analyses, serum-based studies showed higher pooled specificity than plasma-based studies. A formal threshold effect assessment did not reveal a statistically significant correlation between sensitivity and false-positive rate (Spearman ρ = −*0.334, p* = *0.062).* Deeks’ test suggested potential small-study effects (p = 0.043).

**Conclusions:**

Circulating microRNA and lipidomic/metabolomic biomarkers demonstrate strong overall diagnostic performance for breast cancer detection; however, substantial heterogeneity and potential small-study effects limit their immediate clinical translation. Given that most included studies used retrospective case-control designs rather than prospective screening cohorts, these biomarkers are best regarded as investigational, complementary tools rather than replacements for mammography at this stage. Future large, prospective, standardized studies with harmonized pre-analytics and prespecified thresholds are needed to support the implementation of screening or triage pathways.

## Introduction

Breast cancer is the most frequently diagnosed cancer among women worldwide and remains a leading cause of cancer-related mortality. Recent global estimates indicate a substantial and continuing burden, with breast cancer responsible for hundreds of thousands of deaths annually and representing a major share of incident cancers in women across most countries. These realities make earlier detection, before regional or distant spread, one of the most impactful strategies for improving outcomes and reducing treatment intensity [1,2].

Mammography-based screening has led to earlier diagnosis at the population level and remains the standard of care recommended by major screening guidelines, although significant clinical constraints remain [3,4]. Dense breast tissue, in particular, may disguise lesions and has been linked to decreased mammographic sensitivity and specificity; screening thus has the potential to generate false-positive results, excessive imaging, and unnecessary biopsies [5,6]. These gaps have accelerated efforts to develop minimally invasive biomarkers that can complement imaging and improve early identification and triage.

Liquid biopsy offers a promising, non-invasive way to detect cancer by measuring tumour-related signals in easily accessible biofluids. Among the most studied candidates are the circulating microRNAs (miRNAs), small, non-coding RNAs that regulate gene expression after transcription. Notably, miRNAs can be detected in plasma or serum in a remarkably stable form, protected from degradation by endogenous RNases, and can be measured reproducibly using PCR-based platforms [7]. A growing body of evidence has linked changes in circulating miRNA profiles with breast cancer [8,9], supporting their potential use for early detection and for multi-marker panels that may better reflect biological heterogeneity than any single analyte, consistent with the broader shift toward multi-omic liquid biopsy strategies in oncology [10].

In parallel, lipidomics has emerged as a powerful approach for capturing tumour-associated metabolic reprogramming. Because lipid metabolism is increasingly recognized as central to cancer biology, tumour-driven alterations in lipid species may generate measurable circulating signatures with diagnostic relevance [11]. Importantly, lipidomic panels measured in plasma have been shown to distinguish between early-stage breast cancer, benign lesions, and healthy controls, indicating the biological plausibility of lipid-derived liquid biopsy signals as complementary diagnostic markers [12].

However, published estimates of diagnostic accuracy for circulating miRNAs and lipidomic biomarkers vary widely across studies due to differences in patient selection, disease stage, control definitions (healthy vs benign), specimen type (serum vs plasma), analytical platforms (qRT-PCR/dPCR vs LC–MS/MS), normalization and cut-off selection, and whether single biomarkers or multi-marker panels are evaluated. The distinction between serum and plasma is not merely nominal: during the clotting process required for serum preparation, platelets and other blood cells release additional miRNAs into the sample, which can raise or otherwise alter measured concentrations relative to plasma, whereas anticoagulated plasma (e.g., EDTA or citrate) better preserves the true circulating profile provided that strict pre-centrifugation protocols are followed to minimize platelet activation and haemolysis [13,14]. Because few studies report matched serum–plasma comparisons or standardized pre-analytical handling, specimen type is a plausible and clinically important source of the heterogeneity observed across studies. Such heterogeneity limits clinical translation and makes it difficult to identify the most reliable biomarker candidates for validation and implementation.

As a result, we conducted this systematic review and meta-analysis in line with PRISMA 2020 to quantitatively evaluate the diagnostic accuracy of circulating miRNAs and lipidomic biomarkers in early breast cancer diagnosis. By pooling key performance measures, sensitivity, specificity, diagnostic odds ratios, and AUC, we aim to identify the most reliable individual biomarkers and multi-marker panels with the strongest potential for clinical translation. We also explore where differences between studies come from by using subgroup and comparative analyses to investigate sources of heterogeneity. Ultimately, these findings are intended to guide future validation work and support the development of integrated, non-invasive biosensing platforms for earlier breast cancer detection

## Materials and methods

This systematic review and meta-analysis were conducted in accordance with the PRISMA guidelines for diagnostic test accuracy studies. A comprehensive literature search was performed in several databases, including MEDLINE (via PubMed), Web of Science, Scopus, Springer, ScienceDirect, and the Cochrane Library, to identify relevant studies published from 2015 to 2025. The search strategy combined terms related to circulating microRNAs, metabolomic or lipidomic biomarkers, and breast cancer diagnosis. No language restrictions were applied. Peer-reviewed full-text articles were eligible regardless of publication language. Two reviewers independently screened all titles and abstracts and then evaluated the full texts of potentially eligible studies. Any discrepancies in study selection were resolved through discussion and consensus (with a third reviewer consulted when needed).

**Handling of non-English studies:** Titles/abstracts published in languages other than English were screened using translation when necessary. For potentially eligible records, full texts were translated to confirm eligibility and extract data. Where a full text could not be translated sufficiently to extract the required diagnostic accuracy outcomes, the record was excluded, and the reason was documented in the PRISMA flow diagram.

**Fig 1:**
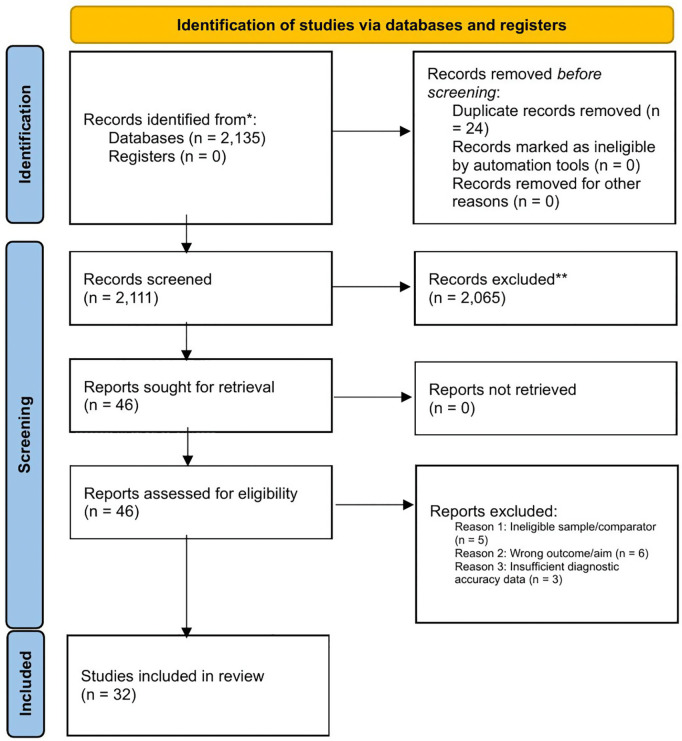
PRISMA 2020 flow diagram illustrating the identification, screening, eligibility assessment, and inclusion of studies evaluating circulating microRNA and lipidomic/metabolomic biomarkers for breast cancer detection.

The systematic literature search identified a total of 2,135 records from electronic databases. After removal of 24 duplicate records, 2,111 unique records were screened based on titles and abstracts. Of these, 2,065 records were excluded for failing to meet the predefined inclusion criteria. A total of 46 full-text articles were assessed for eligibility. Fourteen studies were excluded due to an ineligible sample or comparator type (n = 5), inappropriate outcome or study aim (n = 6), or insufficient diagnostic accuracy data (n = 3). Ultimately, 32 studies fulfilled all eligibility criteria and were included in the quantitative synthesis and meta-analysis (Fig 1).

### Inclusion and exclusion criteria

We included studies that evaluated the diagnostic accuracy of circulating microRNA-based assays or metabolomic/lipidomic biomarker panels for detecting breast cancer (particularly early-stage disease) in humans. Eligible studies were required to report (or provide data to calculate) the numbers of true positives, false positives, true negatives, and false negatives, from which sensitivity and specificity could be derived. Both prospective cross-sectional studies and retrospective case–control designs were eligible. Studies focusing on biomarkers measured in whole blood (unseparated blood samples) were excluded to maintain consistency in specimen type. If multiple publications analyzed the same patient cohort, only the most comprehensive or recent report was included to avoid duplicate data.

### Data extraction and quality assessment

From each included study, data were extracted on study characteristics (publication year, design), sample size (number of breast cancer cases and controls), specimen type (plasma, serum, urine, etc.), the index test(s) used (e.g., specific microRNAs or metabolites), and the diagnostic performance outcomes (sensitivity, specificity, or contingency table values). When a study did not directly provide a full contingency table, we reconstructed the 2 × 2 table of outcomes – i.e., true positives (TP), false negatives (FN), false positives (FP), and true negatives (TN) – using the reported sensitivity, specificity, and sample size of cases and controls. A continuity correction of 0.5 was applied to any cell with zero counts during this reconstruction to enable calculation of log-odds and variances. Two researchers independently performed the data extraction, and any conflicts were resolved by review of the original article.

The methodological quality of each study was appraised using the QUADAS-2 tool (Quality Assessment of Diagnostic Accuracy Studies 2). This assessment evaluated potential bias in four domains: patient selection, index test, reference standard, and flow of patients/timing of procedures. Each domain was rated as having low, high, or unclear risk of bias according to QUADAS-2 guidelines, and the applicability of the study to the review question was also judged. Quality assessment was conducted by two reviewers working independently, with disagreements resolved through consensus.

### Ethical considerations

Ethical approval was not required for this systematic review and meta-analysis, as it involved only the retrieval and synthesis of aggregate, study-level data from previously published reports (secondary data analysis); no primary patient data, identifiable information, or new human or animal experimentation were involved.

### Statistical analysis

We used meta-analytic techniques to synthesize the diagnostic accuracy measures across studies. For each study, sensitivity and specificity proportions were transformed using the logit function, and random-effects meta-analyses (DerSimonian–Laird method) were performed to calculate pooled estimates of sensitivity and specificity with corresponding 95% confidence intervals (CIs). This approach accounts for between-study variability in effect sizes. In addition, we calculated the summary diagnostic odds ratio (DOR) for the index tests as an overall indicator of diagnostic performance. Pooled positive and negative likelihood ratios (PLR and NLR) were derived either from the summary sensitivity and specificity or by pooling individual study log likelihood ratios under a random-effects model. Heterogeneity across studies was quantified using the *I^2^ statistic* (with values of ~50% indicating moderate heterogeneity) and assessed with Cochran’s Q test. We also calculated between-study variance (τ^2^) as part of the random-effects model output.

Pre-specified subgroup analyses were carried out to explore potential sources of heterogeneity. We stratified studies by the biological specimen used for the biomarker assay (plasma vs. serum vs. urine) to compare diagnostic accuracy across specimen types. Owing to inconsistent or incomplete reporting of analytical platform (e.g., qRT-PCR vs. NGS vs. LC-MS/GC-MS), patient geographic region/ethnicity, disease stage (early-stage vs. mixed-stage), biomarker class (circulating microRNA-only vs. lipidomic/metabolomic vs. combined panels), and marker-panel size across the primary studies, these additional stratifications could not be robustly or uniformly performed with the data as reported and are flagged as priorities for a future individual participant data (IPD) meta-analysis. We also conducted a univariable meta-regression to investigate time trends in test accuracy: publication year of the study was examined as a moderator to determine if more recent studies reported different sensitivity or specificity than earlier studies. The meta-regression was performed on the logit-transformed sensitivity (as the dependent variable) against study year. To assess the possibility of a threshold effect, whereby variation in implicit diagnostic cut-offs across studies drives an inverse relationship between sensitivity and specificity, we calculated the Spearman correlation coefficient between the logit-transformed sensitivity and logit-transformed false-positive rate (1 − specificity) across all included studies. We also derived Youden’s Index (J = sensitivity + specificity − 1) for each study and for the pooled estimates, as a single summary measure of overall diagnostic performance that is independent of disease prevalence.

To evaluate the potential for publication bias and small-study effects, we constructed a funnel plot of the log (DOR) versus its standard error for the included studies. Deeks’ test was applied to quantify funnel plot asymmetry, as Deeks’ test is the recommended approach for diagnostic accuracy meta-analyses because it accounts for the mathematical coupling between the diagnostic odds ratio and its standard error that can otherwise produce spurious asymmetry with Egger’s test [15]. A significant p-value indicated the presence of small-study effects. Finally, we performed sensitivity analyses to test the robustness of the meta-analysis results. We conducted leave-one-out analysis in which the meta-analysis was repeated, omitting one study at a time; this checked whether any single study had an undue influence on the pooled estimates. All statistical analyses were performed using Python’s *stats models* library and supplementary routines, with results reported as two-sided p-values where applicable, using R 4.4.2 and Python 3.12.1. All code and scripts are publicly available on GitHub: https://github.com/swatian1989.

## Results

### Study characteristics

The systematic search yielded 2,135 records, of which 2,111 unique citations were screened after duplicate removal. Following title and abstract screening, 46 full-text articles were assessed for eligibility. Fourteen studies were excluded due to inappropriate sample or comparator types (n = 5), irrelevant outcomes or study aims (n = 6), or insufficient diagnostic accuracy data (n = 3). Ultimately, 32 studies fulfilled all inclusion criteria and were incorporated into the quantitative synthesis (Fig 1).

The included studies were published between 2015 and 2025 and comprised a total of 6,935 participants, including 3,697 patients with breast cancer and 3,238 non-cancer controls. Sample sizes ranged from 40 participants (20 cases/20 controls) to 1,042 participants (540 cases/502 controls). Most investigations employed case–control designs and primarily evaluated circulating microRNA-based diagnostic panels, while a smaller subset assessed lipidomic or metabolomic signatures.

Biological specimens included plasma (n = 17), serum (n = 6), EDTA plasma (n = 3), extracellular vesicle–enriched plasma or serum fractions (n = 3), mixed plasma/serum designs (n = 2), and urine-derived exosomes (n = 1). All studies reported sufficient data to reconstruct 2 × 2 contingency tables (true positives, false positives, false negatives, and true negatives). Internal consistency checks confirmed concordance between reported case/control numbers and extracted diagnostic counts. A continuity correction was applied where zero-cell counts were present. Detailed study characteristics and diagnostic performance metrics are summarized in Table 1.

**Table 1:** Characteristics and diagnostic performance of studies evaluating circulating microRNA and lipidomic/metabolomic biomarkers for breast cancer detection.

Authors (Reference)	Year	Study Title	Specimen Type	Breast Cancer Cases (n)	Controls (n)	Sensitivity	Specificity	AUC	Youden’s Index (J)
**Mardi et al.** [16]	2025	Experimental Validation of miR-4443, miR-572, and miR-150-5p in Serum and Tissue of Breast Cancer Patients as a Potential Diagnostic Biomarker	Serum	26	26	0.914	0.814	0.918	0.728
**Jing et al.** [17]	2024	*Diagnostic value of 5 mi*RNA*s combined detection for breast cancer*	Serum	90	65	0.750	0.826	0.866	0.576
**Li et al.** [18]	2024	*Discovery of Plasma Lipids as Potential Biomarkers Distinguishing Breast Cancer Patients from Healthy Controls*	Plasma	298	300	0.821	0.893	0.913	0.714
**Liu et al.** [19]	2023	Discovery of Lipid Profiles in Plasma-Derived Exosomes for Breast Cancer Diagnosis	Plasma (EVs)	105	54	0.825	0.800	0.880	0.625
**Kumar et al.** [20]	2023	Impact of three miRNA signature as potential diagnostic marker for **triple negative breast cancer** patients	Serum	139	51	0.908	0.815	0.919	0.723
**Giordano et al.** [21]	2023	miRNAs in the Box: Potential Diagnostic Role for Extracellular Vesicle-Packaged miRNA-27a and miRNA-128 in Breast Cancer	Serum (EVs)	45	36	0.851	0.839	0.908	0.690
**El-Toukhy et al.** [22]	2023	The diagnostic significance of circulating miRNAs and metabolite profiling in early prediction of breast cancer in Egyptian women	Serum	30	20	1.000	1.000	1.000	1.000
**Zhao et al.** [23]	2022	A circulating miR-19b-based model in diagnosis of human breast cancer	Plasma (EDTA)	120	50	0.723	0.734	0.792	0.457
**Zou et al.** [24]	2022	Development and validation of a circulating microRNA panel for the early detection of breast cancer	Serum	540	502	0.851	0.785	0.887	0.636
**Mohmmed et al.** [25]	2021	A Clinical Evaluation of Circulating MiR-106a and Raf-1 as Breast Cancer Diagnostic and Prognostic Markers	Plasma / serum	50	30	0.813	0.906	0.903	0.719
**Itani et al.** [26]	2021	A Signature of Four Circulating microRNAs as Potential Biomarkers for Diagnosing Early-Stage Breast Cancer	Plasma	41	32	0.906	0.744	0.862	0.650
**Liu et al.** [27]	2021	Cross-platform genomic identification and clinical validation of breast cancer diagnostic biomarkers	Serum (± exosomes)	224	113	0.860	0.823	0.907	0.683
**Diansyah et al.** [28]	2021	Early Detection of Breast Cancer: The Role of Circulating MicroRNAs	Plasma	26	16	0.923	0.812	0.923	0.735
**Canatan et al.** [29]	2021	*Circulating micro*RNA*s as Potential Non-invasive Biomarkers for Breast Cancer Detection*	Plasma	20	20	0.833	0.824	0.856	0.657
**Adam-Artigues et al.** [30]	2021	Identification of a Two-MicroRNA Signature in Plasma as a Novel Biomarker for Very Early Diagnosis of Breast Cancer	Plasma	72	163	0.778	0.689	0.762	0.467
**Lopes et al.** [31]	2021	miR-210 and miR-152 as Biomarkers by Liquid Biopsy in Invasive Ductal Carcinoma	Plasma	30	15	0.927	0.818	0.931	0.745
**Jang et al.** [32]	2021	Multiple microRNAs as biomarkers for early breast cancer diagnosis	Plasma	226	146	0.917	0.817	0.934	0.734
**Wolrab et al.** [33]	2021	Plasma lipidomic profiles of kidney, breast and prostate cancer patients differ from healthy controls	Plasma	103	192	0.876	0.897	0.937	0.773
**Ibrahim et al.** [34]	2020	Candidate circulating microRNAs as potential diagnostic and predictive biomarkers for the monitoring of locally advanced breast cancer patients	Plasma	30	20	0.719	0.938	0.870	0.657
**Savitri et al.** [35]	2020	Circulating Plasma miRNA-21 as a Superior Biomarker Compared to CA 15−3: Assessment in Healthy Age Matched Subjects and Different Stage of Breast Cancer Patients	Plasma (EDTA)	49	16	0.800	0.850	0.890	0.650
**Ashirbekov et al.** [36]	2020	Combination of circulating miR-145-5p/miR-191-5p as biomarker for breast cancer detection	Plasma	35	33	0.943	1.000	0.984	0.943
**Guo et al.** [37]	2020	Plasma miR-1273g-3p acts as a potential biomarker for early Breast Ductal Cancer diagnosis	Plasma	39	40	0.884	0.725	0.842	0.609
**Hirschfeld et al.** [38]	2020	Urinary Exosomal MicroRNAs as Potential Non-invasive Biomarkers in Breast Cancer Detection	**Urine (exosomal)**	69	40	0.946	0.925	0.980	0.871
**Li et al.** [39]	2018	Circulating microRNAs from the miR‑106a–363 cluster on chromosome X as novel diagnostic biomarkers for breast cancer	Plasma & serum	320	400	0.893	0.857	0.930	0.750
**Li et al.** [40]	2019	A five‐miRNA panel in plasma was identified for breast cancer diagnosis	Plasma	257	257	0.854	0.878	0.913	0.732
**Shaheen et al.** [41]	2019	Identification of Circulating miRNAs as Non- Invasive Biomarkers of **Triple Negative Breast** Cancer in the Population of Pakistan	Plasma	37	34	0.833	0.870	0.907	0.703
**Yuan et al.** [42]	2019	A plasma metabolite panel as biomarkers for early primary breast cancer detection	Plasma (EDTA)	189	150	0.817	0.844	0.879	0.661
**Swellam et al.** [43]	2018	Role of some circulating MiRNAs on breast cancer diagnosis	Serum	80	70	0.788	0.773	0.846	0.561
**Fang et al.** [44]	2019	Plasma MicroRNA Pair Panels as Novel Biomarkers for Detection of Early-Stage Breast Cancer	Plasma	53	78	0.958	0.705	0.879	0.663
**Jové et al.** [45]	2017	A plasma metabolomic signature discloses human breast cancer	Plasma	91	20	0.733	0.733	0.806	0.466
**Frères et al.** [46]	2015	Circulating microRNA-based screening tool for breast cancer	Plasma	149	133	0.647	0.794	0.719	0.441
**Matamala et al.** [47]	2015	Tumor MicroRNA Expression Profiling Identifies Circulating MicroRNAs for Early Breast Cancer Detection	Plasma	114	116	0.868	0.737	0.828	0.605

**AUC**: Area under the receiver operating characteristic curve; **EVs**: Extracellular vesicles; **EDTA**: Ethylenediaminetetraacetic acid.

Across studies, reported diagnostic performance was generally high, with most assays achieving sensitivities and specificities exceeding 0.75. Multi-marker microRNA panels and lipidomic models consistently demonstrated superior performance compared with single-biomarker approaches, with several studies reporting AUC values above 0.90. Youden’s Index (J), calculated as sensitivity + specificity − 1 for each study, ranged from 0.441 to a maximum of 1.000, with most studies clustering between 0.6 and 0.75, reflecting generally strong but variable overall discriminative performance across the included biomarker panels.

### Overall diagnostic accuracy

Random-effects meta-analysis demonstrated strong pooled diagnostic performance of circulating microRNA and lipidomic/metabolomic biomarkers for breast cancer detection. The pooled sensitivity was 0.87 (95% CI [0.83, 0.90]), indicating that approximately 87% of breast cancer cases were correctly identified. The pooled specificity was 0.84 (95% CI [0.79,0.88]), corresponding to accurate classification of nearly 84% of non-cancer controls.

The pooled diagnostic odds ratio (DOR) was 46.10 (95% CI [27.80, 76.70]), reflecting a high level of overall discrimination between cases and controls. The pooled positive likelihood ratio (PLR) was 5.17 (95% CI [3.99, 6.68]), suggesting that a positive test result increased the post-test probability of breast cancer more than fivefold. Conversely, the pooled negative likelihood ratio (NLR) was 0.16 (95% CI [0.13, 0.21]), indicating a substantial reduction in disease probability following a negative test result. The pooled Youden’s Index (J), a prevalence-independent summary measure combining sensitivity and specificity, was 0.71 (95% CI [0.65, 0.77]), reflecting strong overall discriminative ability of circulating microRNA and lipidomic/metabolomic biomarkers for breast cancer detection. Summary diagnostic accuracy estimates are presented in Table 2.

**Table 2:** Pooled diagnostic accuracy estimates for circulating microRNA and lipidomic/metabolomic biomarkers derived from random-effects meta-analysis.

Metric	Pooled Estimate	95% CI
Sensitivity	0.87	[0.83, 0.90]
Specificity	0.84	[0.79, 0.88]
Diagnostic odds ratio (DOR)	46.10	[27.80, 76.70]
AUC (SROC)	0.92	[0.87, 0.94]
Positive likelihood ratio (PLR)	5.17	[3.99, 6.68]
Negative likelihood ratio (NLR)	0.16	[0.13, 0.21]
Youden’s Index (J)	0.71	[0.65, 0.77]

### Forest plot analysis

**Fig 2:**
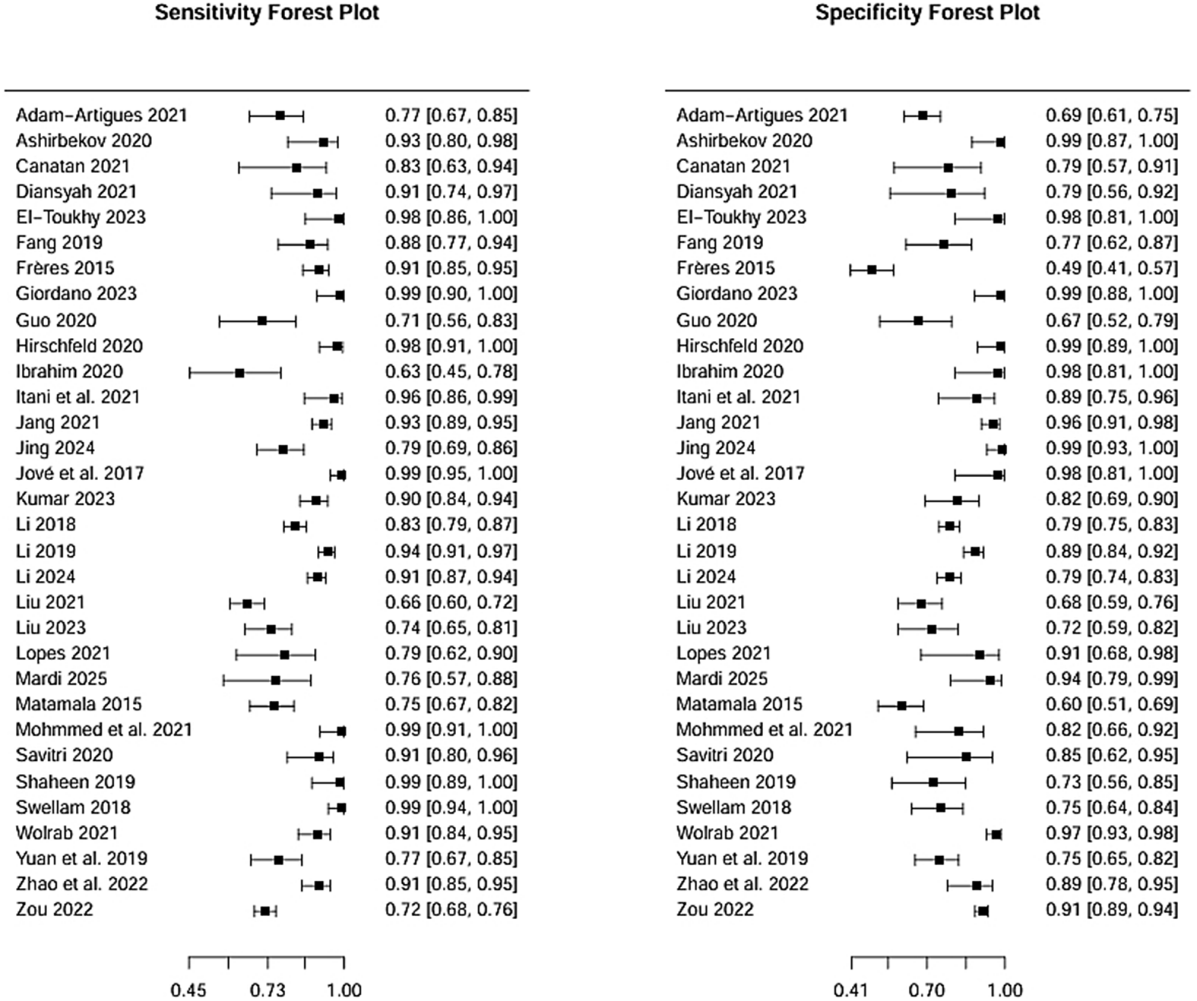
Forest plots of sensitivity and specificity for circulating microRNA and lipidomic/metabolomic biomarkers across included studies. Squares represent study-specific estimates, horizontal lines indicate 95% confidence intervals, and diamonds denote pooled estimates from random-effects models.

Forest plots illustrating individual-study sensitivity and specificity estimates are shown in Fig 2. Substantial variability was observed across studies; however, the majority reported sensitivity and specificity values above 0.75. Despite this heterogeneity, the pooled estimates remained stable, as indicated by the narrow confidence intervals around the summary effects, supporting the robustness of the meta-analytic findings.

### Summary receiver operating characteristic (SROC) analysis

**Fig 3:**
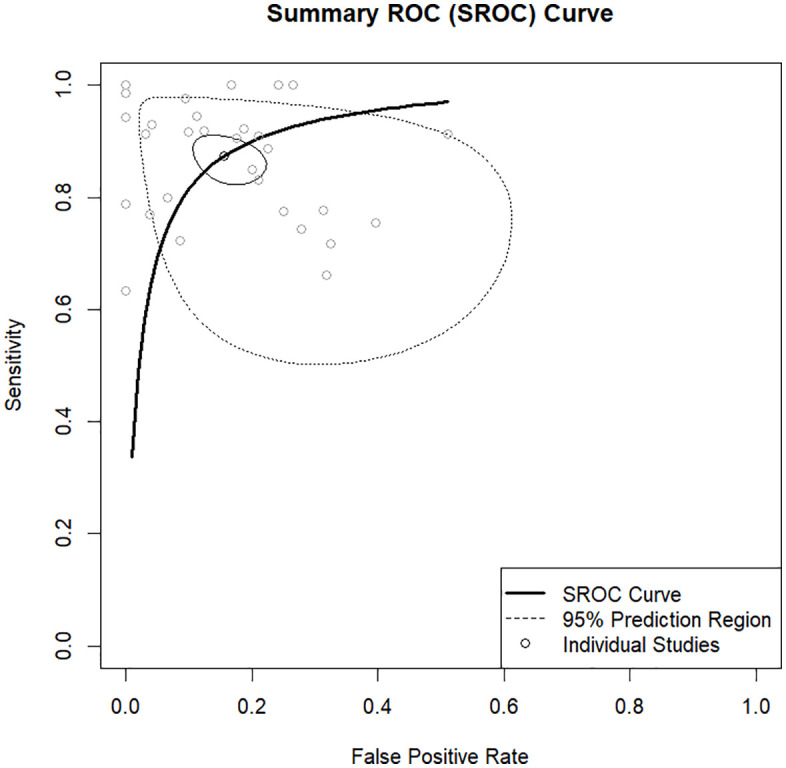
Summary receiver operating characteristic (SROC) curve for circulating microRNA and lipidomic/metabolomic biomarkers in breast cancer detection, with 95% confidence and prediction regions.

The summary receiver operating characteristic (SROC) curve is presented in Fig 3. The curve is positioned close to the upper-left corner of the ROC space, indicating a favorable trade-off between sensitivity and specificity across studies. The pooled area under the curve (AUC) was 0.92 (95% CI [0.87, 0.94]), consistent with excellent overall diagnostic accuracy.

### Diagnostic odds ratio across included studies

**Fig 4:**
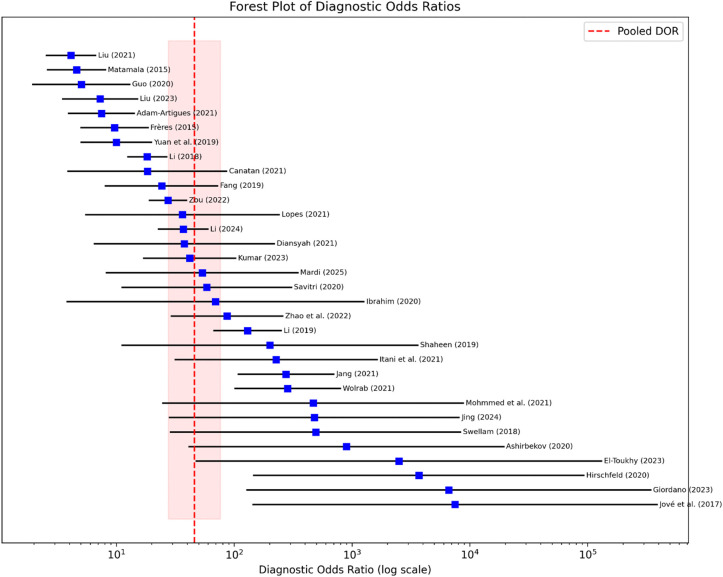
Forest plot of diagnostic odds ratios (DORs) for individual studies assessing circulating microRNA and lipidomic/metabolomic biomarkers, together with the pooled DOR estimated using a random-effects model.

Individual-study diagnostic odds ratios exhibited marked variability, as shown in Fig 4. Nevertheless, the random-effects model yielded a high pooled DOR of 46.10 (95% CI [27.80, 76.70]), confirming strong discriminatory performance across diverse study settings. Wider confidence intervals were observed in smaller studies, reflecting increased uncertainty, but no single study disproportionately influenced the pooled estimate.

### Heterogeneity assessment

Considerable between-study heterogeneity was detected. Sensitivity demonstrated high heterogeneity (*Q*(31) = 222.20, *p* < 0.0001; *I^2^* = 88.29%), and specificity showed similarly elevated heterogeneity (*Q*(31) = 251.20, *p* < 0.0001; *I^2^* = 90.20%). Heterogeneity was also substantial for ln (DOR) (*I*^2^ = 93.01%) and logit-transformed AUC estimates (*I*^2^ = 94.09%) (Table 3).

**Table 3:** Between-study heterogeneity statistics for pooled diagnostic accuracy outcomes estimated using random-effects models.

Outcome	*k*	τ^2^ (SE)	τ	*I*^*2*^ (%)	*H* ^*2*^	Q(df)	*p*(Q)
Sensitivity (logit)	32	0.6080 (0.2097)	0.7797	88.29	8.54	222.196 [31]	<0.0001
Specificity (logit)	32	0.7814 (0.2669)	0.8840	90.20	10.21	251.201 [31]	<0.0001
Ln (DOR)	32	2.6226 (0.8498)	1.6195	93.01	14.31	275.839 [31]	<0.0001
AUC (logit; approx.)	32	1.0818 (0.3282)	1.0401	94.09	16.93	388.039 [31]	<0.0001

*Note. k* = number of studies; τ^2^ = between-study variance; τ = √τ^2^; *I^2^* = proportion of total variability due to heterogeneity; *H^2^* = heterogeneity ratio; *Q* = Cochran’s *Q* statistic.

Given the expected methodological and clinical diversity among studies—including differences in biomarker composition, analytical platforms, specimen handling, threshold selection, and control definitions—a random-effects model was prespecified. Subgroup analysis was conducted to explore potential sources of heterogeneity, particularly specimen type.

### Subgroup analysis by specimen type

Subgroup analysis restricted to studies with clearly defined specimen categories (*k* = 25) was summarized in Table 4. Plasma-based assays (*k* = 16) yielded a pooled sensitivity of 0.88 (95% CI [0.83, 0.92]) and a pooled specificity of 0.84 (95% CI [0.74, 0.90]). Serum-based studies (*k* = 6) demonstrated comparable sensitivity, 0.89 (95% CI [0.71, 0.96]), but higher pooled specificity, 0.91 (95% CI [0.79, 0.97]). Studies using EDTA plasma (*k* = 3) showed pooled sensitivity and specificity of 0.88 (95% CI [0.76, 0.94]) and 0.83 (95% CI [0.71, 0.91]), respectively.

**Table 4:** Subgroup analysis of pooled diagnostic accuracy estimates stratified by biological specimen type.

Specimen	*k*	Pooled sensitivity	95% CI	Pooled specificity	95% CI
Plasma	16	0.88	[0.83, 0.92]	0.84	[0.74, 0.90]
Serum	6	0.89	[0.71, 0.96]	0.91	[0.79, 0.97]
Plasma (EDTA)	3	0.88	[0.76, 0.94]	0.83	[0.71, 0.91]

*Note. k* = number of studies; CI = Confidence intervals.

Overall, pooled sensitivities were broadly consistent across specimen types, whereas serum-based assays tended to exhibit higher specificity. Formal statistical comparisons between subgroups were not performed due to limited study numbers within strata.

### Meta-regression analysis

**Fig 5:**
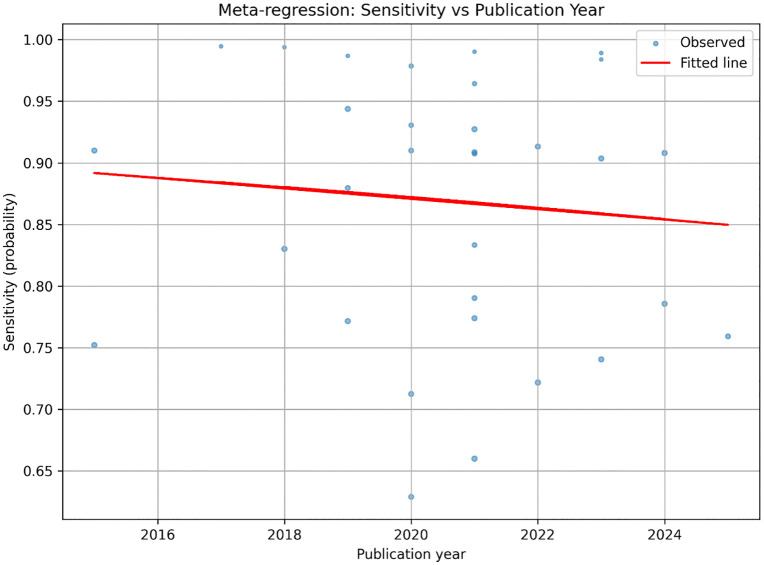
Meta-regression analysis of diagnostic sensitivity by year of publication. Each circle represents an individual study, with circle size proportional to study weight. The solid line indicates the fitted regression line.

Meta-regression analysis using publication year as a moderator did not reveal a significant temporal trend in diagnostic sensitivity (*p* = 0.62) (Fig 5). This finding suggests that reported sensitivity has not systematically increased or decreased over time. A parallel meta-regression for specificity likewise showed no statistically significant temporal trend, consistent with the sensitivity findings reported above.

### Assessment of publication bias and small-study effects

**Fig 6:**
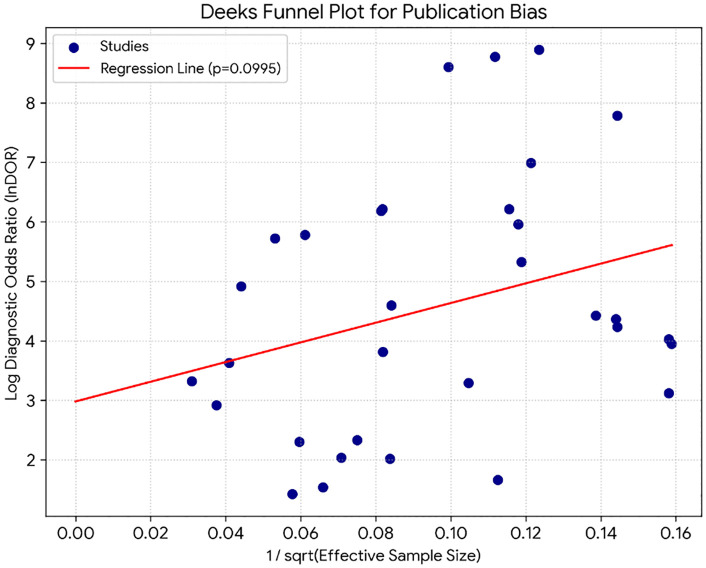
Deeks’ funnel plot assessing small-study effects in diagnostic accuracy studies of circulating microRNA and lipidomic/metabolomic biomarkers for breast cancer detection.

**Fig 7:**
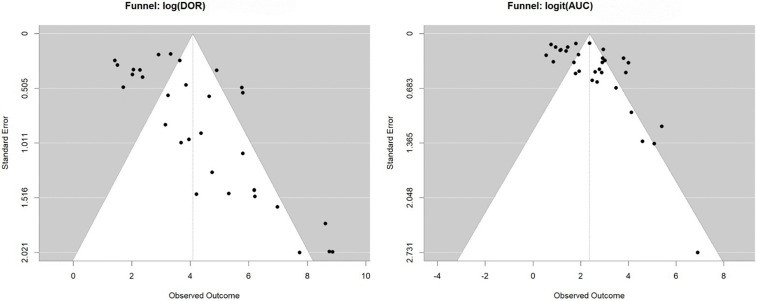
Funnel plot evaluating potential publication bias in studies assessing the diagnostic performance of circulating microRNA and lipidomic/metabolomic biomarkers for breast cancer detection.

Potential small-study effects and publication bias were evaluated using funnel plot–based methods (Figs 6 and 7). Visual inspection of the funnel plots revealed asymmetry, suggesting that smaller or less precise studies may report disproportionately higher diagnostic accuracy estimates. This observation was statistically supported by Deeks’ funnel plot asymmetry test (*p* = 0.043), indicating the presence of small-study effects. Collectively, these findings suggest that the pooled diagnostic performance may be partially influenced by publication bias and therefore should be interpreted with appropriate caution.

### Threshold effect analysis

To assess whether a threshold effect contributed to the observed heterogeneity, we examined the Spearman correlation between the logit-transformed sensitivity and logit-transformed false-positive rate (1−specificity) across studies. This correlation was moderate and approached but did not reach conventional statistical significance (ρ = −0.334, p = 0.062), providing suggestive but inconclusive evidence that variation in implicit diagnostic thresholds across studies may have contributed to the observed heterogeneity. Given the borderline p-value and moderate effect magnitude, a genuine threshold effect cannot be excluded; threshold-related heterogeneity and possible threshold overfitting remain plausible contributors that merit direct examination with future access to raw participant-level data (Table 5).

**Table 5:** Threshold effect assessment.

Assessment	Statistic	p-value
Spearman correlation (logit Sensitivity vs logit FPR)	rho = −0.3341287	0.06162

*Note.* FPR: false-positive rate

### Methodological quality and risk of bias assessment

**Fig 8:**
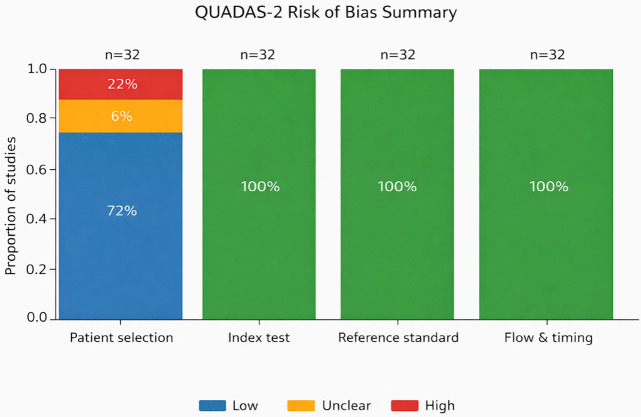
QUADAS-2 risk-of-bias and applicability assessment for included studies, presented as traffic-light plots across the four domains: patient selection, index test, reference standard, and flow and timing.

Assessment using the QUADAS-2 tool revealed that most studies were at low risk of bias for the index test and reference standard domains (Fig 8). However, patient selection and flow/timing domains frequently exhibited unclear or high risk of bias, primarily due to case–control designs, non-consecutive recruitment, and incomplete reporting. Overall, methodological quality was judged to be moderate, highlighting the need for large, prospective diagnostic accuracy studies with standardized protocols.

## Discussion

This systematic review and meta-analysis evaluated the diagnostic accuracy of circulating microRNAs and lipidomic/metabolomic biomarkers for early breast cancer detection across 32 studies comprising 6,935 participants. The analysis revealed strong overall diagnostic performance, with a pooled sensitivity of 0.87 (95% CI [0.83, 0.90]), a specificity of 0.84 (95% CI [0.79, 0.88]), and an SROC AUC of 0.92 (95% CI [0.87, 0.94]). The pooled diagnostic odds ratio (DOR) of 46.10 (95% CI [27.80, 76.70]) indicates that circulating molecular biomarkers substantially increase the odds of correctly identifying breast cancer cases compared to controls. The positive likelihood ratio (PLR) of 5.17 suggests that a positive test result meaningfully increases the post-test probability of disease, while the negative likelihood ratio (NLR) of 0.16 indicates that a negative test substantially reduces the likelihood of breast cancer. These findings position circulating microRNAs and lipidomic biomarkers as promising candidates for non-invasive early breast cancer detection, potentially complementing existing screening modalities.

The diagnostic performance observed in this meta-analysis aligns with and extends previous systematic reviews examining circulating biomarkers for breast cancer detection. Earlier meta-analyses of circulating microRNAs reported a pooled sensitivity of 0.85 and specificity of 0.83 with SROC AUCs between 0.89 and 0.91 [48]. Our findings fall within the upper range of these estimates, likely reflecting both the inclusion of more recent studies with refined methodologies and the incorporation of lipidomic biomarkers alongside microRNA panels. The combined analysis of both microRNA and lipidomic markers may capture complementary biological information, potentially explaining the enhanced diagnostic performance observed.

Compared to conventional screening mammography, which demonstrates a sensitivity of approximately 0.85 to 0.90 in average-risk populations but shows reduced performance (0.65, 0.75) in women with dense breast tissue [5,8], the pooled sensitivity and specificity observed for circulating microRNA and lipidomic/metabolomic biomarkers in this meta-analysis were numerically similar. However, because the large majority of included studies used retrospective case–control designs rather than prospective screening cohorts, these estimates are likely to overestimate real-world screening performance, and a direct comparison with mammography is not appropriate at this stage; these biomarkers should instead be regarded as investigational, complementary tools rather than as potential replacements for imaging. This distinction is particularly relevant given that 40–50% of screening-age women have heterogeneously or extremely dense breasts, where mammographic sensitivity is substantially compromised [6]. The low NLR (0.16) observed in these case–control settings is a promising signal for a potential rule-out role, but this application would need to be confirmed in prospective, screening-representative populations before any claim of reduced unnecessary biopsies following false-positive mammographic findings could be made.

Recent studies have explored multi-omics integration for breast cancer detection, combining genomic, transcriptomic, proteomic, and metabolomic data [49,50]. While such comprehensive approaches may theoretically enhance diagnostic accuracy, they also increase complexity, cost, and analytical requirements. Our findings suggest that focused panels of circulating microRNAs and lipid metabolites can achieve strong diagnostic performance, potentially offering a more pragmatic balance between accuracy and feasibility for clinical implementation.

The strong diagnostic performance of circulating microRNAs reflects their fundamental roles in breast cancer biology. MicroRNAs regulate post-transcriptional gene expression and are dysregulated in breast tumorigenesis, affecting pathways involved in cell proliferation, apoptosis, epithelial-mesenchymal transition, and metastasis [51]. Tumor cells actively secrete microRNAs into circulation via exosomes, microvesicles, and protein complexes, making them detectable in blood at early disease stages [52]. The stability of circulating microRNAs, conferred by their packaging in extracellular vesicles or binding to RNA-binding proteins, enables their measurement in clinical specimens with acceptable pre-analytical variability [13].

Similarly, lipidomic alterations in breast cancer reflect fundamental metabolic reprogramming in malignant cells. Cancer cells exhibit altered lipid metabolism to support rapid proliferation, requiring increased synthesis of membrane phospholipids, signaling lipids, and energy storage molecules [11]. Specific lipid species, including phosphatidylcholines, sphingomyelins, ceramides, and lysophospholipids have been consistently associated with breast cancer presence and progression [12]. These lipid alterations are detectable in circulation, likely reflecting both tumor-derived lipids and systemic metabolic responses to malignancy. The integration of microRNA and lipidomic biomarkers may capture both regulatory (microRNA) and metabolic (lipid) dimensions of breast cancer biology, potentially explaining their combined diagnostic utility.

The diagnostic performance metrics observed in this meta-analysis suggest several potential clinical applications. Circulating biomarker panels could serve as adjunctive tools to improve breast cancer detection in populations where mammography performance is suboptimal, particularly women with dense breasts, younger women (age < 50 years), or those at intermediate risk who may not qualify for supplemental MRI screening [3,4]. The high sensitivity (0.87) suggests that biomarker testing could reduce false-negative screening results, while the high specificity (0.84) indicates acceptable control of false-positive findings.

Biomarker panels could also function as triage tools following abnormal mammographic findings: with a positive likelihood ratio of 5.17, a positive biomarker result would meaningfully increase post-test probability of malignancy and may help guide the urgency and intensity of follow-up imaging or biopsy decisions, whereas a negative likelihood ratio of 0.16 indicates that a negative result could support identification of lower-risk abnormalities suitable for short-interval surveillance rather than immediate biopsy, potentially reducing overdiagnosis and overtreatment. In addition, the minimally invasive nature of blood-based testing could enable more frequent monitoring in high-risk populations such as women with BRCA1/BRCA2 mutations or strong family histories complementing annual MRI and mammography and supporting interval testing to detect cancers arising between scheduled imaging [2,10]. Finally, in resource-limited settings where access to mammography is constrained, validated circulating biomarker assays could potentially serve as primary screening tools to identify women who should be prioritized for imaging evaluation; however, this would require prospective validation in representative populations alongside careful evaluation of cost-effectiveness, assay standardization, and health-system readiness.

The substantial heterogeneity observed in this meta-analysis (*I*^2^ = 88.29% for sensitivity; 90.20% for specificity) is a critical limitation that warrants careful interpretation, as multiple methodological and clinical factors likely drive between-study variability. Pre-analytical differences were prominent, with studies using different specimen matrices (serum vs. plasma) and heterogeneous collection, processing, and storage procedures; because circulating microRNA stability and lipidomic profiles are sensitive to time-to-processing, centrifugation conditions, freeze–thaw cycles, and storage duration, inconsistent handling can meaningfully alter measured signals and downstream accuracy estimates [14,53]. The subgroup finding of higher specificity in serum-based studies compared with plasma further suggests that matrix effects contribute to performance differences, potentially reflecting cellular microRNA release during clotting and compositional differences in lipid fractions between matrices.

Analytical heterogeneity also likely contributed: studies employed diverse platforms for miRNA profiling (qRT-PCR, microarray, next-generation sequencing) and lipidomic (LC–MS, GC–MS), each with distinct sensitivity, dynamic range, and reproducibility characteristics, alongside variable normalization strategies (e.g., reference controls/spike-ins for miRNAs and internal standards/transformations for lipids), which can shift both absolute values and classifier behavior [54]. Biomarker selection varied substantially across studies, spanning individual miRNAs, multi-miRNA panels, lipid species, and combined multi-marker signatures; because multi-marker models can capture biological heterogeneity more effectively than single analytes, differences in panel composition and modeling approaches plausibly translate into divergent diagnostic performance, yet sparse reporting limited robust subgroup comparisons of single-marker versus multi-marker strategies.

Study design and population characteristics likely amplified heterogeneity, as the predominance of case–control designs can overestimate diagnostic accuracy relative to prospective cohort designs through spectrum effects and differential verification bias [55,56]. Control group definitions varied, including healthy volunteers, women with benign breast disease, or mixed populations, potentially affecting specificity estimates. Breast cancer case characteristics, including stage distribution, histological subtypes (luminal A, luminal B, HER2-enriched, triple-negative), and grade, varied across studies and may influence biomarker expression patterns. The predominance of early-stage disease in included studies is appropriate given the focus on early detection, but limits generalizability to more advanced cancers.

Finally, threshold selection differed widely, including pre-specified cutoffs, data-driven “optimal” thresholds, and algorithmic classifiers; thresholds derived and tested within the same dataset can overestimate performance due to overfitting [57]. The lack of standardized, externally validated thresholds across studies contributes to heterogeneity and complicates clinical translation.

The Deeks’ funnel plot asymmetry test was significant (*p* = 0.043), indicating potential small-study effects and/or publication bias, and this signal is plausibly multifactorial. Selective publication is a leading explanation, whereby studies reporting statistically significant or clinically striking diagnostic accuracy are more likely to appear in the published literature, while null or modest findings remain unpublished, an issue repeatedly documented in diagnostic test accuracy research [15,58]. This risk may be accentuated here, given the relatively small median sample size and the broad spread of study sizes, which increases the influence of small, “positive” studies on pooled estimates. Methodological factors may also contribute, as smaller studies can be more prone to design and conduct limitations such as inadequate blinding, selective reporting of candidate biomarkers, and post-hoc threshold optimization, potentially inflating apparent diagnostic accuracy [55]. While the QUADAS-2 assessment was performed, residual confounding by unmeasured quality dimensions may persist. Finally, in the context of substantial heterogeneity, asymmetry may reflect heterogeneity-related artifacts rather than publication bias alone; if study size is correlated with underlying differences in patient spectrum, control selection, assay platform, or analytical strategy, then true performance differences linked to these features can generate size-dependent effects that mimic publication bias [59].

The detection of potential small-study effects suggests that the pooled diagnostic accuracy estimates may be optimistic and should be interpreted cautiously. Larger, prospective, pre-registered studies with comprehensive reporting regardless of results are needed to provide more robust estimates.

## Conclusions

This systematic review and meta-analysis indicate that circulating microRNA and lipidomic/metabolomic biomarkers have strong overall diagnostic performance for breast cancer detection (pooled sensitivity 0.87, specificity 0.84; SROC AUC 0.92). Collectively, these blood-based molecular signatures show promise as minimally invasive, investigational adjuncts that could complement mammography, particularly in settings where imaging performance is challenged (e.g., dense breasts) or where improved risk refinement and triage could reduce unnecessary downstream procedures; they are not intended as a replacement for imaging-based screening.

Nonetheless, the current evidence base does not support immediate clinical adoption. Substantial between-study heterogeneity (I^2^ > 88%), evidence of small-study effects (Deeks’ *p* = 0.043), and recurring methodological limitations, including predominance of case–control designs, heterogeneous biomarker panels and analytical platforms, and limited standardization of pre-analytical handling, normalization, and diagnostic thresholds, raise the likelihood that pooled estimates overstate performance relative to real-world screening pathways. Because screening populations have lower disease prevalence and greater clinical heterogeneity, accuracy observed under controlled research conditions may not translate directly to routine practice.

Future work should prioritize harmonized pre-analytical/analytical standards; large, prospective validation in true screening or pathway-embedded cohorts using pre-specified panels and locked thresholds; richer reporting of clinical and pathological features to enable clinically meaningful subgroup analysis; and comparative effectiveness studies testing biomarker-enhanced strategies against standard screening. Health-economic evaluations and clearly defined regulatory/clinical validation pathways will also be essential for translation. With rigorous standardization and prospective confirmation, circulating microRNA and lipidomic biomarkers may contribute to more personalized screening approaches that improve early detection while minimizing false positives and unnecessary interventions; until then, they should be regarded as investigational and not yet ready for routine clinical implementation.

## Strengths and limitations

### Strengths

This systematic review and meta-analysis have several methodological strengths. A comprehensive, multi-database search strategy across six major databases maximized capture of the available evidence. Reporting was structured according to PRISMA 2020, and the protocol was prospectively registered in PROSPERO, strengthening transparency and helping to mitigate selective reporting. Methodological quality was appraised rigorously using QUADAS-2, enabling a structured assessment of risk of bias and applicability across diagnostic accuracy studies. Pooled estimates of sensitivity and specificity were generated using logit-transformed, random-effects (DerSimonian–Laird) modelling, a well-established approach for synthesizing diagnostic accuracy data across heterogeneous studies. In addition, the use of SROC visualization alongside a full set of diagnostic performance indices (sensitivity, specificity, likelihood ratios, DOR, Youden’s Index, and AUC) provided a clinically interpretable and comprehensive characterization of test performance. Finally, planned subgroup analysis and assessment of small-study effects using Deeks’ test enhanced the interpretability and strengthened the contextualization of the findings.

### Limitations

Significant heterogeneity across studies (*I*^2^ > 88%) limits confidence in pooled estimates, and residual variability persisted despite subgroup analysis. The predominance of case–control designs introduce potential spectrum and verification bias, likely inflating diagnostic accuracy compared with screening settings. Variation in control group definitions further restricts generalizability. Evidence of small-study effects and publication bias (Deeks’ *p* = 0.043) suggests possible overestimation of performance. Additionally, heterogeneity in biomarkers, analytical platforms, and a lack of methodological standardization hinder the comparability and clinical translation of results. Finally, diagnostic accuracy alone does not establish clinical utility, underscoring the need for prospective, outcome-focused studies.

## Supporting information

S1 Checklist PRISMA 2020 checklist for this systematic review and meta-analysis. (DOCX)

S1 File Graphical Abstract. (TIF)

## Abbreviations

AUC: area under the receiver operating characteristic curve
BC: breast cancer
CI: confidence interval
DOR: diagnostic odds ratio
EDTA: ethylenediaminetetraacetic acid
EVs: extracellular vesicles
FN: false negative
FP: false positive
I^2^: inconsistency index (measure of heterogeneity)
LC–MS: liquid chromatography–mass spectrometry
LC–MS/MS: liquid chromatography–tandem mass spectrometry
miRNA (miR): microRNA
NLR: negative likelihood ratio
PLR: positive likelihood ratio
PRISMA: Preferred Reporting Items for Systematic Reviews and Meta-Analyses
qRT-PCR: quantitative reverse transcription polymerase chain reaction
QUADAS-2: quality assessment of diagnostic accuracy studies 2
ROC: receiver operating characteristic
SROC: summary receiver operating characteristic
TN: true negative
TP: true positive
τ^2^: between-study variance
WHO: World Health Organization
